# 3-Dimensional histological reconstruction and imaging of the murine pancreas

**DOI:** 10.1007/s00335-014-9522-2

**Published:** 2014-05-18

**Authors:** Steven L. Ciciotte, Mark Lessard, Ellen C. Akeson, Elizabeth Cameron, Timothy M. Stearns, James M. Denegre, Jesus Ruberte, Karen L. Svenson

**Affiliations:** 1 The Jackson Laboratory, Bar Harbor, ME 04609 USA; 2 Center of Animal Biotechnology and Gene Therapy, Department of Animal Health and Anatomy, Universitat Autònoma de Barcelona, Barcelona, Spain

## Abstract

Visualization of important disease-driving tissues in their native morphological state, such as the pancreas, given its importance in glucose homeostasis and diabetes, provides critical insight into the etiology and progression of disease and our understanding of how cellular changes impact disease severity. Numerous challenges to maintaining tissue morphology exist when one attempts to preserve or to recreate such tissues for histological evaluation. We have overcome many of these challenges and have developed new methods for visualizing the whole murine pancreas and single islets of Langerhans in an effort to gain a better understanding of how islet cell volume, spatial distribution, and vascularization are altered as diabetes progresses. These methods are readily adaptable without requirement for costly specialized equipment, such as magnetic resonance imaging, positron emission tomography, or computed tomography, and can be used to provide additional robust analysis of diabetes susceptibility in mouse models of Type 1 and Type II diabetes.

**Electronic supplementary material:**

The online version of this article (doi:10.1007/s00335-014-9522-2) contains supplementary material, which is available to authorized users.

## Introduction

The hallmark of diabetes, whether autoimmune (Type I; T1D) or otherwise acquired (Type II; T2D, gestational), lies in dysfunction of the pancreatic β-cell to respond appropriately in the management of circulating blood glucose. Decades of research in diabetes have markedly advanced our understanding of the etiology of diabetic conditions and continue to contribute to the development of effective strategies for treatment. Investigation of diabetes in the laboratory has been greatly facilitated by the use of rodents to model features of human diabetes in concert with technological improvements in methodologies used to evaluate these models both in vitro and in vivo. Challenges remain in understanding how to translate histopathological observations to the assessment of pancreatic function both from exocrine and endocrine perspectives. One way to improve the evaluation of the diabetic state in rodent models is to visualize the intact pancreas and its islets of Langerhans. This has been partially achieved in previously reported strategies (Weaver 1989; Berclaz et al. 2012), although these methodologies are not readily adaptable to laboratories that lack specialized equipment to prepare and analyze samples.

There is strong clinical relevance of monitoring of islet degradation, β-cell mass, and vasculature changes during progression of diabetes, with equal need for these studies in murine models, as clinical symptoms in humans and mouse may not present until as much as 60–80 % of β-cell mass has been compromised (Cnop et al. 2005). Non-invasive imaging methods would be ideal for facilitating rapid analysis and efficacy of treatments in both the clinical and research settings. Current in vivo techniques such as magnetic resonance imaging and positron emission tomography are effective for labeling and following islet transplantation, and molecular identification of specific targets, respectively, but lack the required resolution for quantitation of islet volume and β-cell mass (Di Gialleonardo et al. 2012). Advances in probe development and resolution continue to drive these approaches, but the instruments are not available to many researchers (Arifin and Bulte 2011).

Optical imaging methods include intra-vital, in vivo and ex vivo approaches, and are most applicable to pre-clinical research. One advantage of these methods is the capability to collect quantitative data in concert with spatial information in the context of the whole pancreas. Optical projection tomography depends on back-projection construction of sample volumes and is most effective when used with fluorescent contrast agents such as antibodies. It has been applied to whole tissue pancreata reconstruction, providing quantitation within the limited resolution and an informative spatial organization (Alanentalo et al. 2010). Optical coherence tomography uses light and interferometry to generate contrast in tissues, without the need for contrast agents, and may be used in unfixed, live tissues (Villiger et al. 2009; Berclaz et al. 2012). Other light methods are based on fluorescence and bioluminescence markers, both transgenically expressed (Yong et al. 2011) and applied post-vivo (Agudo et al. 2012). Bioluminescent imaging is effective at detecting 10-fold differences in expression, but lacks the sensitivity for in-depth morphological analysis. Recent development of fluorescent probes, in conjunction with intra-vital microscopy, improves capabilities in quantitation (Reiner et al. 2011).

In the absence of strongly effective, or readily accessible, in vivo β-cell and islet quantitative imaging, improvements in histological approaches can achieve comprehensive quantitation of islet volume, β-cell volume, and extent and organization of vascularization around islets.

The origin of 3-D reconstructions from serial sections was developed at the end of the nineteenth century for the study of human embryos (His 1880). However, it was not until early in the 1970s when computerized techniques were sufficiently well developed to assist such work and 3-D modeling of microscopic structures became possible (Levinthal and Ware 1972). Initially, 3-D reconstructions were made manually, but with the passage of time and great progress in computer imaging, semi-automatic techniques were introduced to make reconstruction less time consuming (Carlbom et al. 1994). This process has been further enhanced with the development of commercially available software, such as Imaris, which can stack the series of cross-sections for 3-D reconstruction. In addition, the use of confocal laser microscopy combined with the broad range of new fluorescent probes and specific computational frameworks provides methods to automatically segment, register, quantify, and reconstruct the 3-D state of microscopic structures (Feng et al. 2005).

This report presents two strategies for visualizing elements of the murine pancreas that will guide functional analysis and enhance our understanding of important mechanisms contributing to the diabetic state. Full reconstruction of the pancreas demonstrates distribution and quantifiable size variability of islets of Langerhans, while visualization of single islets with intact vascularization offers insight to the interdependence of endocrine cells and blood vessels.

## Materials and methods

Pancreata were harvested independently for use in one of the two visualization strategies: full reconstruction of whole pancreas and reconstruction of single islets. Methods followed for each strategy are presented below.

### Animals and tissue harvest for whole pancreas reconstruction

Three inbred strains used in this study [C57BL/6J (B6), NODShiLt/J (NOD), CAST/EiJ (CAST)] were obtained from The Jackson Laboratory, Bar Harbor, Maine. We also used an ENU-derived mutant (HLB62) generated in our laboratory (KLS) that is heterozygous for a splice acceptor mutation in the glucokinase gene (*Gck*) and is therefore moderately hyperglycemic. Pancreata were harvested from three male mice per strain, aged 12–14 weeks, fed a standard chow diet (LabDiet^®^ 5K52, St. Louis, MO, USA). Mice were euthanized by cervical dislocation. Harvested tissues were placed in Bouin’s fixative for 24 h, then run through graded alcohols, xylene, and molten paraffin. Bouin’s fixative best preserves morphology and subsequent Aldehyde Fuchsin protocols (below). Tissues were embedded in paraffin, serially sectioned at 6 µm, and placed on glass slides.

### Histological staining

After sectioning, slides were deparaffinized and rehydrated. To increase contrast of islets and vasculature from exocrine tissue, the B6 and HLB62 pancreata were stained with Gomori’s Aldehyde Fuchsin. The CAST and NOD samples were stained using a modified Aldehyde Fuchsin protocol. In the modified procedure, slides were placed into Lugol’s iodine for 30 min, washed in water, and placed into 5 % sodium thiosulfate for 2 min. Slides were then rinsed in water, followed by a rinse in 70 % ethanol, and immersed in Aldehyde Fuchsin for 30 min. Excess stain was removed by rinsing in 95 % ethanol followed by a water wash. Slides were then immersed into Light Green/Orange G for 45 s to counterstain, rinsed in 0.2 % acetic acid, dehydrated, cleared, and coverslipped.

### Imaging for full reconstruction

Slides were digitally scanned using a Nanozoomer 2.0HT (Hamamatsu). Individual sections were automatically cropped from the .ndpi files using Glom v3.1 (Jackson Laboratory, Computational Sciences), a custom-written program for batch processing of tissue sections on slides. Individual section images were then processed through an automated pipeline consisting of a series of thresholding and masking steps written in CellProfiler to remove extraneous material outside the tissue area. Processed images were then run through a second CellProfiler pipeline utilizing the “UnmixColors” feature to isolate the individual colors produced by the staining methods. When the colors are segmented in Unmix, the resulting images are in black and white. By this method, the Aldehyde Fuchsin and Light Green/OrangeG stains provide preferential islet staining, preferential blood vessel staining, and a general background tissue staining.

Individual images from the separated colors were then assembled in image stacks that were organized top-to-bottom by image section number. This resulted in three image stacks for each pancreas: one for the islet staining; one for the vessel staining; and one for the whole tissue staining. Although the image stacks are in the correct order, each section is not registered to those around it. Using the MultiStackReg1.45 plugin in FIJI, we performed a rigid registration of the whole tissue image stack and recorded the transformation matrix that was calculated. This matrix was then loaded and applied to the other two image stacks (islet staining, vessel staining) for that pancreas. Once all three image stacks had completed the registration step, they were combined to create one image stack containing all three color channels. This final image stack was then loaded into Imaris 7.6.5 (Bitplane), although any reconstruction program may be used. The 3-D data were thresholded to the boundaries of the tissue to create a visual boundary for each pancreas. Using the surface creation tool in Imaris, surfaces were put on the boundaries of the tissues generated for each of the three channels, and relevant statistics were exported for analysis, allowing for volume determination. Parameters obtained were ellipticity (oblate and prolate), sphericity, volume and area for both individual islets and for the pancreas as a whole. Because our interest was in parameters describing 3-dimensional features, we excluded area (µm^2^) from our analysis and focused on the analysis of volume (µm^3^). Fiji and CellProfiler are freely available software products.

### Statistical analysis of islet volume from full reconstruction parameters

An analysis of variance was used to determine if strain had a significant effect (*p* value <0.05) on predicting islet volume. Since several hundred technical replicates were gathered per sample, the median islet volume (of technical replicates) was used as a representative volume for the comparison. Since strain was identified to have a significant effect on islet volume, a Tukey HSD post hoc test was used to identify significant differences between strains. Given the large range between minimum and maximum islet volume (volume difference of millions), the islet volume measurements (of technical replicates) of C57BL/6J biological replicates (control samples) were combined, and used to identify four bins of islet volume as designated by each quartile (bin 1 represents islet volume below the first quartile measurement, bin 2 represents islet volume above or equal to the first quartile measure but below the median, bin 3 represents islet volume above the median but below or equal to the third quartile measurement, and bin 4 represents islet volume above or equal to the third quartile measurement). The technical replicates for each sample were placed in one of the four bins based on islet volume, and the percent of the total replicates per bin was calculated per sample. An additional analysis of variance was used to determine if strain had a significant effect on percentage per bin. Since strain was identified to have a significant effect on the percentage per bin for the fourth bin only, a Tukey HSD post hoc test identified significant differences between CAST and C57BL/6J, and CAST and NOD.

### Single islet reconstruction: animals and tissue harvest

Pancreata were harvested from six male C57BL/6J mice, aged 12 weeks, fed a standard chow diet. Mice were euthanized by anesthetic overdose. To harvest the pancreas, the thoracic aorta was cannulated and the abdomen perfused with 10 % neutral buffered formalin. After careful dissection of the whole pancreas, islets and their surrounding exocrine tissue were excised under a stereoscopic microscope (Nikon SMZ800). By reflecting light, white pancreatic islets were easily distinguished surrounded by the ivory colored exocrine acini. Pancreatic pieces of 1 mm^3^ were permeabilized overnight at 4 °C with 0.1 % Triton X-100 (Sigma, St. Louis, MO).

### Pancreas immunohistochemistry and visualization for single islet analysis

Primary incubation was performed overnight at 4 °C with guinea pig anti-insulin (1:100) (Abcam, Cambridge, UK) and rabbit anti-collagen IV (1:200) (Millipore, Temecula, CA), both diluted in wash buffer (phosphate-buffered saline supplemented with 0.3 % bovine serum albumin). Normal goat serum (10 %) was added to the primary antibody to ensure minimum background staining. The secondary antibodies were goat anti-guinea pig Alexa Fluor 488 (1:100) (Invitrogen, Carlsbad, CA) and goat anti-rabbit Alexa Fluor 568 (1:100) (Invitrogen). The incubation was performed at 4 °C overnight. Nuclei were counterstained with bisbenzimide Hoechst 33342 (1:1000; Sigma). After immunolabeling, specimens were mounted in excavated slides with antiphotobleaching medium (Sigma). Islets were localized by their green fluorescence and a series of confocal sections every 1 µm were made with the laser scanning confocal microscope (TCS SP2; Leica Microsystems GmbH, Heidelberg, Germany). Fluorescent signal was obtained with three simultaneous confocal detection channels. Insulin signal localized in pancreatic β cells was visualized using an argon laser (488 nm). Blood vessels inside the islets were observed using a HeNe laser (594 nm) that excited Alexa 568, previously bound to the collagen IV localized in the basement membrane. Nuclei stained with Hoechst were recorded using a 405 nm laser.

### Plasma glucose, insulin, and glucose tolerance testing

Blood samples were obtained from the retro-orbital sinus after administration of topical anesthetic (tetracaine HCl) using a heparin-coated microcapillary tube and collected into 1.5 ml Eppendorf tubes. Approximately 150 µl of whole blood was collected into tubes containing 2 µl 10 % sodium heparin, and plasma was separated by centrifugation at 10,000 rpm for 10 min at 4 °C and removed into a clean Eppendorf tube. Blood for glucose measurements was collected after food was removed from animals for four hours in the morning and samples were analyzed using the Beckman Synchron DXC600Pro Clinical Chemistry analyzer. Animals were not fasted for insulin measurements and blood was drawn in a separate event >2 weeks after prior blood sampling. Insulin was measured using the Meso Scale Discovery Mouse/Rat Insulin kit (Meso Scale Discovery, Rockville, MD). Glucose tolerance testing was conducted after mice were fasted overnight for 16 h. The following morning mice were weighed to calculate dose required to administer 2 mg glucose/g body weight intraperitoneally. Prior to glucose administration, mice were gently restrained to score tail tip with a lancet for blood collection. An initial sample was taken for baseline analysis, followed immediately by bolus administration. Additional samples were obtained at 15, 30, 60, 120, and 180 min after glucose injection. Samples were analyzed using the Abbott AlphaTRAK Glucometer using a fresh test strip at each time point.

## Results

### Diabetic state of sampled animals

**Fig. 1:**
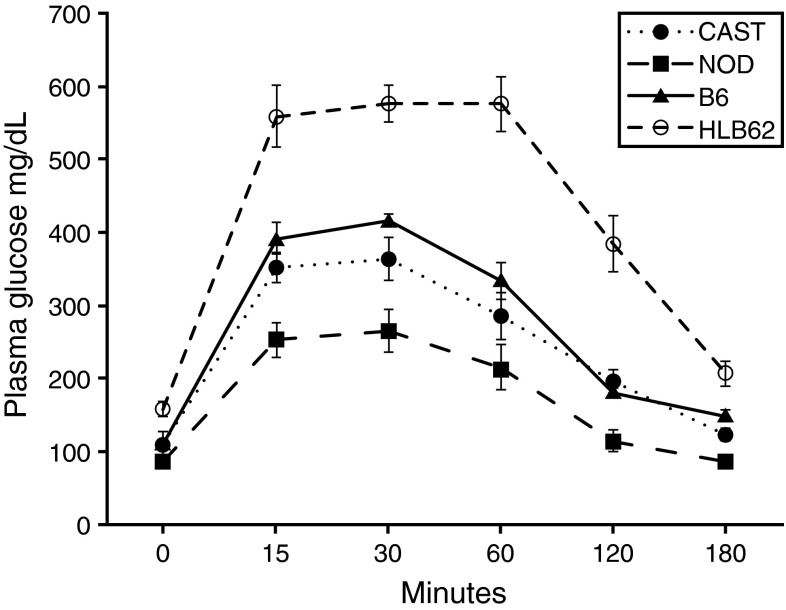
Intra-peritoneal glucose tolerance test for each strain used in this study. *X*-axis is minutes after glucose bolus was injected. *N* = 5–8 males per strain

Plasma glucose and insulin values for strains used in this study are presented in Table 1. It is notable that CAST has the highest circulating plasma insulin although the strain is not considered hyperinsulinemic or insulin resistant. This may simply reflect a more aggressive management of glucose homeostasis. The *Gck* mutant showed delayed glucose clearance in the glucose tolerance test while CAST, NOD, and B6 strains did not (Fig. 1). Hence the *Gck* mutant males were the only overtly diabetic mice used in this study. The hyperglycemia exhibited by the *Gck* mutant may also be a result of lack of glucose storage in tissues (Wang et al. 2013).

**Table 1:** Plasma glucose and insulin for strains used in this study

Strain	Glucose (mg/dl)	Insulin (ng/µl)
C57BL/6J	201 ± 46	0.64 ± 0.54
CAST/EiJ	187 ± 37	2.4 ± 2.1
NOD/ShiLtJ	156 ± 41	1.63 ± 0.78
HLB62 (*Gck*)	334 ± 42	1.81 ± 0.57

Values are presented as average ± standard deviation. Glucose values were obtained after mice were fasted for 4 h in the morning. Insulin values were obtained from non-fasted animals. Values are for males aged 12–14 weeks; *n* = 5–8 animals per strain

### Comparison of histological slides using two β-cell staining methods

**Fig. 2:**
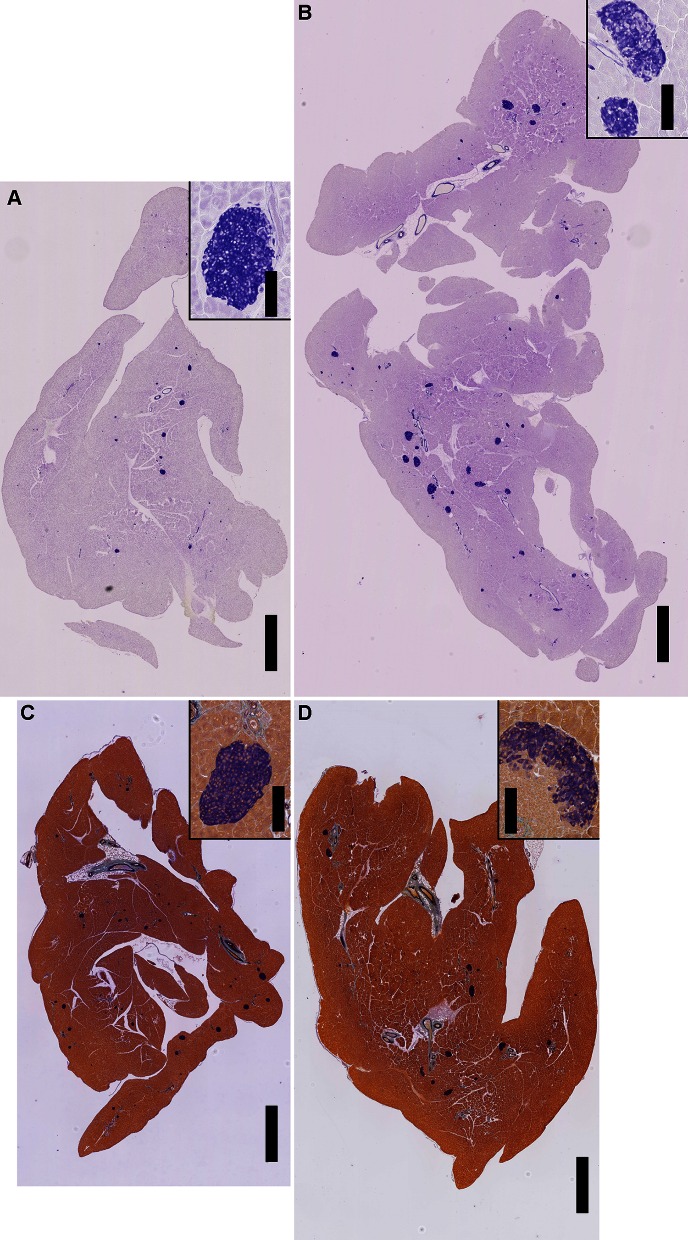
Histological sections of pancreata stained to identify insulin-producing cells. **a** C57BL/6 J; **b** *Gck* mutant HLB62; **c** CAST/EiJ; **d** NOD/ShiLtJ. Full pancreas images are at ×2 magnification and include a 1 mm black scale bar; insets of individual islets per strain are at ×20 magnification and include a 100 µm black scale bar. Samples in **c** and **d** were prepared using the modified staining method

Representative sections of serially-cut whole pancreas for each strain are displayed in Fig. 2. Sections from strains B6 and HLB62 were stained using a standard method for identification of insulin-producing cells and sections from strains CAST and NOD were stained using the modified method. Insets on each panel in Fig. 2 show individual islets. It is notable that, although NOD did not demonstrate measures of overt diabetes from ipGTT, many islets in NOD males are accompanied by surrounding and infiltrating cells, as can be seen in the inset to Fig. 2d. The nuclear morphology of the infiltrating cells is consistent with the inflammatory mononuclear cell infiltrate that is characteristic of the NOD mouse strain and comprised of lymphocytes and macrophages (O’Reilly et al. 1991). The modified method includes a counter stain that allows better discrimination of islet non-β cell endocrine cell types at higher magnification (data not shown), improved nuclear definition, and identification of infiltrating leukocytes.

### Full pancreas reconstruction

**Fig. 3:**
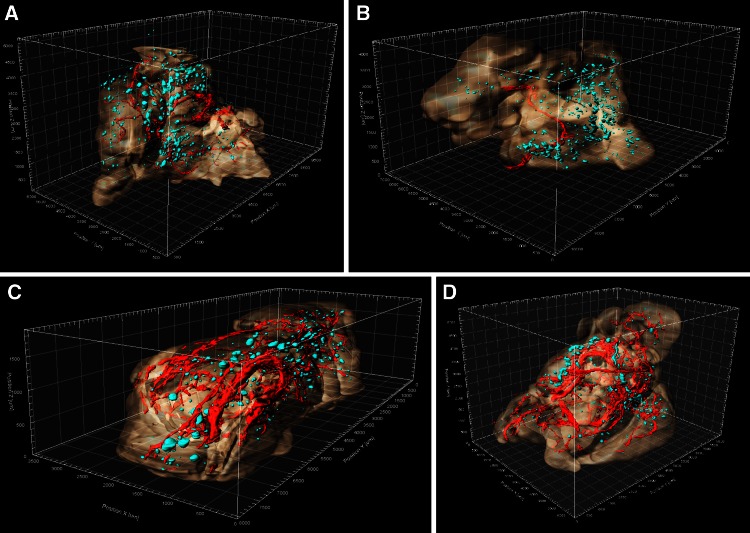
Still images extracted from 3-dimensional reconstructed pancreata from age-matched male mice. **a** C57BL/6J, sample B6-1; **b** *Gck* mutant HLB62, sample HLB62-2; **c** CAST/EiJ, sample CAST-2; **d** NOD/ShiLtJ, sample NOD-2. Tissue boundaries were created using the surface creation tool in Imaris (see “Materials and methods” section). Islets are *blue* and vasculature is *red*

Images of full pancreas reconstructions for animals of each strain are shown in Fig. 3. Animations of 3-D reconstructions from one animal per strain are in Supplemental animations 1–4. While vasculature is prominent in these images, its topographical relations are not maintained. This is due in part to the fact that the pancreatic anatomical portions, right and left lobes and body, were folded in the histological cassette in order to perform the paraffin sections. However, the relationship between great pancreatic vessels and neighboring islets has likely been maintained. Even in the absence of precise vessel topography, there are notable differences in both size and density of the vasculature among the four strains (Fig. 3). Moreover, these images allow both qualitative evaluation of islet distribution throughout the pancreatic regions and quantitative evaluation of the broad variation in islet volume within a single specimen. Table 2 presents quantitative parameters obtained from this analysis and demonstrates wide variability in islet volume and shape for all samples used in the study. Additionally, whole pancreas volumes also vary widely within and among strains (Table 2). Statistical analysis of these values revealed only that CAST values are significantly lower than in other strains. Body weight of CAST mice at 12–14 weeks of age is also significantly less than the other strains (CGD 2009) hence the smaller pancreatic volume is consistent with this difference in total body weight. No significant differences among the other strains were found for islet or whole pancreas volume. It is apparent that failure to detect significant differences among strains is due to high standard deviations of mean values for all strains. To overcome this, we stratified the data for non-diabetic control strain B6 into phenotypic quartile bins describing islet volume as low, medium-low, medium-high, or high. Once the distribution for B6 was established, we compared the other strains to this standard. This analysis (Table 3) revealed that CAST differs significantly from NOD (*p* = 0.012) and B6 (*p* = 0.042) in number of the largest islets (4th quartile bin), demonstrating a lack of large islets in CAST mice. Differences among strains were not found for any other quartiles. The low numbers of biological replicates we used have reduced the statistical power of this analysis and would be improved by adding samples within each strain.

**Table 2:** Islet volume, shape, and total pancreas volume per animal used for full pancreatic reconstruction

Animal	Average islet volume (mm^3^)	Islet volume Min	Islet volume Max	Average islet ellipticity^a^	Average islet sphericity^b^	Average pancreas volume (µm^3^ × 10^9^)	Number of serial sections obtained
B6-1	549.40 ± 57.04	10.09	13029.0	0.32 ± 0.1	0.95 ± 0.07	16.9	756
				0.35 ± 0.1			
B6-2	423.42 ± 41.75	9.86	12430.5	0.37 ± 0.1	0.90 ± 0.1	62.0	523
				0.37 ± 0.1			
B6-3	524.52 ± 44.89	8.27	8821.01	0.37 ± 0.1	0.91 ± 0.1	56.1	488
				0.37 ± 0.1			
HLB62-1	433.82 ± 35.30	17.65	8513.67	0.39 ± 0.1	0.98 ± 0.1	82.3	516
				0.41 ± 0.1			
HLB62-2	496.96 ± 46.58	5.07	7823.37	0.37 ± 0.1	0.92 ± 0.1	98.1	496
				0.39 ± 0.1			
CAST-1	213.49 ± 15.60	9.59	2427.9	0.41 ± 0.2	0.86 ± 0.1	5.0	488
				0.35 ± 0.1			
CAST-2	163.06 ± 20.88	1.69	3979.13	0.39 ± 0.1	0.93 ± 0.1	17.0	382
				0.36 ± 0.1			
CAST-3	413.02 ± 52.63	10.92	8215.72	0.33 ± 0.1	0.95 ± 0.1	14.5	318
				0.33 ± 0.1			
NOD-1	507.57 ± 56.97	20.23	9446.26	0.37 ± 0.1	0.94 ± 0.1	40.6	315
				0.36 ± 0.1			
NOD-2	660.13 ± 81.36	19.20	17770.0	0.37 ± 0.1	0.91 ± 0.1	70.6	379
				0.38 ± 0.1			
NOD-3	1405.79 ± 117.48	179.26	12922.2	0.41 ± 0.2	0.89 ± 0.1	69.8	219
				0.38 ± 0.1			

Values are in mm^3^; average volume is presented ± SEM. Only two animals were analyzed for HLB62

^a^Ellipticity is reported on a scale from 0 to 1, where 0 is a perfect sphere, 0.5 is a perfect ellipse (oblate or prolate), and 1 is a perfect spheroid (oblate or prolate). Values are average ± SD for oblate (top number per cell) and prolate (lower number per cell) orientations

^b^Sphericity is reported on a scale from 0 to 1, where 0 is a linear structure and 1 is a perfect sphere. Values are average ± SD for oblate (top number per cell) and prolate (lower number per cell) orientations

**Table 3:** Quartile analysis of pancreatic islet volume

Strain	Quartile 1^b^ *x* < 25 %	Quartile 2 25 % ≤ *x* < 50 %	Quartile 3 50 % ≤ *x* < 75 %	Quartile 4 *x* ≥ 75 %	*p*-Value v. CAST^c^ (Quartile 4)
Bin range^a^	*x* < 43,206	43,206–125,010	125,010–446,875	*x* ≥ 446,875	n/a
B6-1	119 (24.90 %)	110 (23.01 %)	121 (25.31 %)	128 (26.78 %)	0.042
B6-2	174 (28.16 %)	162 (26.21 %)	146 (23.62 %)	136 (22.01 %)	
B6-3	103 (21.06 %)	124 (25.36 %)	129 (26.38 %)	133 (27.20 %)	
HLB62-1	87 (16.76 %)	161 (31.02 %)	142 (27.36 %)	129 (24.86 %)	n.s.
HLB62-2	149 (29.98 %)	115 (23.14 %)	116 (23.34 %)	117 (23.54 %)	
CAST-1	171 (34.97 %)	135 (27.61 %)	120 (24.54 %)	63 (12.88 %)	n/a
CAST-2	194 (50.65 %)	88 (22.98 %)	74 (19.32 %)	27 (7.05 %)	
CAST-3	94 (29.47 %)	74 (23.20 %)	86 (26.96 %)	65 (20.38 %)	
NOD-1	62 (19.62 %)	90 (28.48 %)	84 (26.58 %)	80 (25.32 %)	0.012
NOD-2	74 (19.47 %)	86 (22.63 %)	104 (27.37 %)	116 (30.53 %)	
NOD-3	112 (23.53 %)	108 (22.69 %)	110 (23.11 %)	146 (30.67 %)	

*n/a* not applicable, *n.s.* non-significant

Strain values were compared to B6 and to each other. Only two animals were analyzed for HLB62. Quartiles are ranked from 1 to 4 by increasing islet volume

^a^Quartile bin ranges were derived from strain B6 values

^b^ *x* denotes a single islet volume per sample; values for samples are number of islets per bin; percentage of all islets per sample contained in quartile is in parentheses

^c^Significant differences at *p* ≤ 0.05 were found only for the 4th bin for CAST compared to B6 and NOD

### Single islet reconstruction

**Fig. 4:**
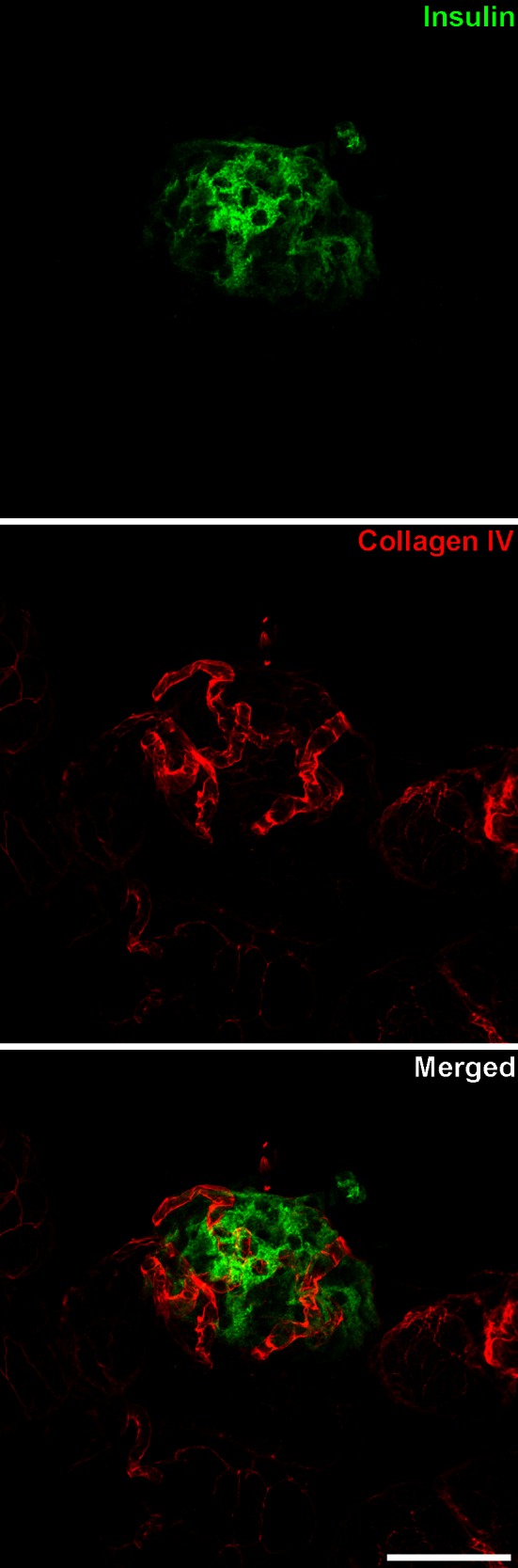
Relationship between insulin-producing cells within a single islet and blood vessels marked with anti-collagen IV can be analyzed in confocal laser microscopy 3D reconstructions. Images are the maximum projection summatory of 31 confocal sections. *Scale bar* 44 µm

**Fig. 5:**
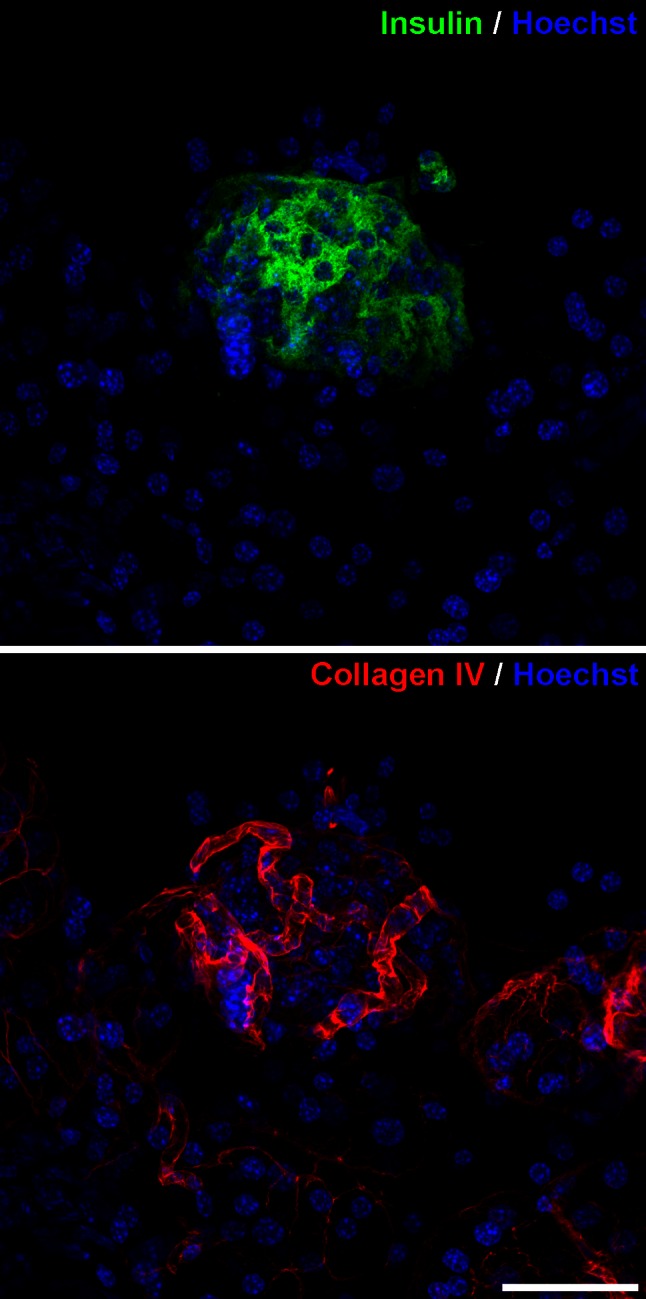
Relationship between an entire pancreatic islet and blood vessels marked with anti-collagen IV can be analyzed in confocal laser microscopy 3D reconstructions using nuclei counterstaining with Hoechst. Images are the maximum projection summatory of 31 confocal sections. *Scale bar* 40 µm

Three-dimensional reconstruction of single islets in strain B6 from a series of 31 confocal laser microscopy sections is shown in Figs. 4 and 5 and in Supplemental animation 5. The relationship and topography between β cells and their vasculature in a single islet are maintained and can be easily demonstrated with this methodology. Collagen IV is present in both the endocrine and the exocrine vascular basement membranes (Figs. 4, 5). With this methodology, we take advantage of the fact that, at the level of individual islets, collagen IV is only found surrounding blood vessels, allowing their unequivocal identification. While insulin-producing β cells are the main component of the pancreatic islets (Fig. 4), other cells such as the glucagon-producing α cells, somatostatin-producing δ cells, and pancreatic polypeptide-producing PP cells are also present in the islet. For this reason, using both nuclei counterstaining and collagen IV identification gives a better understanding of the blood vessels topography inside the entire islet (Fig. 5). Non-insulin-producing islet cells are mainly located at the periphery in murine islets and they can be further revealed using specific antibodies against the specific hormones.

## Discussion

We describe methods for in-depth analysis of mouse models to better understand the nature of islet cell distribution and morphology and how this information can be used to describe the diabetic state. We chose male mice because in diabetic models other than the T1D NOD mouse, males are generally more susceptible to diabetes and other features of the metabolic syndrome than their female counterparts within engineered or natural models of these disorders (Stubbins et al. 2012). Certainly this methodology will also be useful for evaluation of T1D models, where females are more susceptible than males. In the case of NOD in our study, the use of males allowed for analysis of a more intact islet population at the age point chosen. Although the *Gck* mutant was not severely diabetic and NOD males were still normoglycemic at the time of tissue harvest, one can imagine the utility of this approach for evaluating more severely diabetic pancreas by recognizing a significantly diminished quantity of insulin-producing cells or even hyperplastic islets throughout the tissue as an improvement to relying on only a few randomly selected histological sections to make this assessment. Information about regional distribution of islets will inform such inferences as to whether islet size and/or density differences in “head” or “tail” lobular regions are correlated to disease progression or severity. Is there preferential loss or gain of islet volume and accompanying changes in vascularization in specific regions of the pancreas that can be attributed to disease state? Furthermore, the analysis of single islets from more severely diabetic models will facilitate evaluation of how changes in vascular integrity of islets are correlated to β cell failure and further validate previous findings suggesting the importance of this relationship in the maintenance of β cell function (Li et al. 2006; Akirav et al. 2011; Agudo et al. 2012).

There are several challenges posed in the visualization and quantification of murine pancreatic islets. Many islets are very small and their distribution throughout the pancreas is highly heterogeneous. We have demonstrated that, using the methods presented in this report, this size and spatial variation is readily captured. Further, the murine pancreas is a complex structure and differs significantly from that of other mammals, including man (Islam 2010; Balla et al. 2013). The mouse pancreas is sparse and extends along the big curvature of the stomach, contacting its thick tail (left lobe) with the spleen, whereas while the human pancreas topography is similar, it is a more compact gland and its tail is the thinner part of the organ. Collagenase enzymatic digestion of pancreatic islets and posterior staining with dithizone, that specifically binds to Zn in β cells, is a well-known and widely used technique to isolate and observe the morphology of single islets (Latif et al. 1988). However, the enzymatic digestion itself may alter the extracellular matrix and could change the morphology of pancreatic islets. The use of confocal laser microscopy in small pieces of pancreatic tissue allows obtaining the 3-D morphology of single islets in a non-invasive manner, thus eliminating the potential for morphological distortion produced in islets by enzymatic digestion. Improvements to our study that will provide consistency in the orientation of sampled pancreata include the use of injected charcoal powder in specific areas of the pancreas. The charcoal is then visible in histological sections and would provide a reference to understand the disposition of the folded pancreas inside the paraffin block. Additionally, a standardized presentation of pancreata embedded with the splenic lobe to the right, duodenal lobe to the left and gastric lobe centered may also be used.

To demonstrate the methodology described in this report, we used small numbers of samples for only a few mouse strains. Future application of this technique will include a time course of disease progression in one or more established models of diabetes, such as the NOD mouse (females), to encompass early age pre-diabetes through the presentation of overt hyperglycemia for evaluation of changes in islet density and vascularization. In this model, there is slow chronic loss of pancreatic insulin until an abrupt decline between 12 and 14 weeks of age (Gaskins et al. 1992). This observation provides an opportunity to correlate parameters obtained from our methods to this change in insulin, especially islet volume, insulitis, vascular status, and spatial distribution of islets. The use of additional animals per cohort will add statistical power to the analysis of quantitative measures provided by the whole pancreas imaging method.

Hence the ability to fully image this tissue as well as focus on the nature of morphologically maintained individual islets and vascularization will allow important comprehensive evaluation of normal and compromised mouse models of metabolic disease and allow us to address key issues still outstanding relative to the progression of both Type 1 and Type II diabetes.

## Electronic supplementary material

Below is the link to the electronic supplementary material.

Supplementary material 1 (MOV 1647 kb)

Supplementary material 2 (MOV 1786 kb)

Supplementary material 3 (MOV 1424 kb)

Supplementary material 4 (MOV 1798 kb)

Supplementary material 5 (MP4 2360 kb)
